# Trends and characteristics of severe road traffic injuries in children: a nationwide cohort study in Japan

**DOI:** 10.1007/s00068-023-02372-z

**Published:** 2023-10-17

**Authors:** Shunichiro Nakao, Yusuke Katayama, Tetsuhisa Kitamura, Tomoya Hirose, Jotaro Tachino, Kenichiro Ishida, Masahiro Ojima, Takeyuki Kiguchi, Yutaka Umemura, Kosuke Kiyohara, Jun Oda

**Affiliations:** 1 Department of Traumatology and Acute Critical Medicine, Osaka University Graduate School of Medicine, 2-15 Yamadaoka, Suita, Osaka 565-0871 Japan; 2 Department of Social and Environmental Medicine, Division of Environmental Medicine and Population Sciences, Osaka University Graduate School of Medicine, Suita, Japan; 3 Traumatology and Critical Care Medical Center, National Hospital Organization Osaka National Hospital, Osaka, Japan; 4 Division of Trauma and Surgical Critical Care, Osaka General Medical Center, Osaka, Japan; 5 Department of Food Science, Faculty of Home Economics, Otsuma Women’s University, Tokyo, Japan

**Keywords:** Japan Trauma Data Bank, Pediatric traffic injury, Temporal trends, Mortality

## Abstract

**Purpose:**

The purpose of this study was to evaluate temporal trends of characteristics of severe road traffic injuries in children and identify factors associated with mortality using a nationwide database in Japan.

**Methods:**

We performed a retrospective analysis of Japan Trauma Data Bank (JTDB) from 2004 to 2018. We included patients with traffic injuries under the age of 18 who were hospitalized. The primary outcome was in-hospital mortality. We evaluated trends in characteristics and assessed factors associated with in-hospital mortality using a logistic regression analysis.

**Results:**

A total of 4706 patients were analyzed. The most common mechanism of injury was bicycle crash (34.4%), followed by pedestrian (28.3%), and motorcycle crash (21.3%). The overall in-hospital mortality was 11.2%. We found decreasing trends in motorcycle crash and in-hospital mortality and increasing trends in rear passenger seats in cars over the 15-year period. The following factors were associated with in-hospital mortality: car crash (aOR 1.69, 95%CI 1.18–2.40), pedestrian (aOR 1.50, 95%CI 1.13–1.99), motorcycle crash (aOR 1.42, 95%CI 1.03–1.95) [bicycle crash as a reference]; concomitant injuries to head/neck (aOR 5.06, 95%CI 3.81–6.79), thorax (aOR 2.34, 95%CI 1.92–2.87), abdomen (aOR 1.74, 95%CI 1.29–2.33), pelvis/lower-extremity (aOR 1.57, 95%CI 1.23–2.00), spine (aOR 3.01, 95%CI 2.02–4.43); and 5-year increase in time period (aOR 0.80, 95%CI 0.70–0.91).

**Conclusions:**

We found decreasing trends in motorcycle crash and in-hospital mortality, increasing trends in rear passenger seats in cars over the 15-year period, and factors associated with in-hospital mortality such as type of mechanisms and concomitant injuries. Strengthening child road safety measures, particularly for rear passenger seats in vehicles, is imperative to enhance our dedication to injury prevention.

**Supplementary Information:**

The online version contains supplementary material available at 10.1007/s00068-023-02372-z.

## Introduction

Road traffic injuries are currently the leading cause of death among children worldwide, and it is an important health issue that can have social consequences [1]. Various measures have been taken in Japan, and it has been 20 years since the Road Traffic Law was revised in 2000 to make child safety seats mandatory for children under 6 years old. The National Traffic Safety Campaign in Japan is held twice a year by the National Police Agency and other organizations also raise awareness of traffic safety for children [2]. According to a 2019 nationwide survey by the Metropolitan Police Department and the Japan Automobile Federation, the national average rate of child seat use was 70.5%, which is increasing, but the percentage of properly installed child seats was only 47.6% [3]. Motorcycle accidents among children often result in severe trauma and are also an important traffic safety issue [4].

Several studies have been reported on pediatric trauma using the Japan Trauma Data Bank (JTDB), a nationwide database of severe trauma in Japan. A report on pediatric severe trauma found that fall was the most common among those aged 5 years and younger, while traffic injury was the most common among those aged 6–10 years and 11–15 years, at 54% and 48%, respectively [5]. Some studies have investigated factors associated with mortality in traffic trauma while riding a motor vehicle in children, while others have investigated differences in injury site patterns between front passenger and rear seat passengers while riding a motor vehicle [6–8]. As mentioned earlier, Japan has been strengthening traffic safety for children, and studies examining trends in pediatric trauma over time have shown a decrease in the proportion of traffic trauma with respect to the mechanism of injury for blunt injuries (from 59% in 2009 to 45% in 2018) [9]. However, there is not enough research on which type of mechanisms need further improvement in severe traffic injuries in children.

The purpose of this study was to evaluate the epidemiology of severe traffic injuries among children in Japan and their trends over time and to identify mechanisms that need further improvement in severe traffic injuries in children.

## Methods

### Study design and setting

We performed a retrospective analysis of JTDB from 2004 to 2018. The study was approved by the institutional ethics committee of Osaka University Graduate School of Medicine (approval no. 16260–3). Informed consent was not required due to the anonymous nature of the data.

The emergency medical system personnel at the scene select the hospital for patient transport based on the severity of the injury and life-threatening conditions. However, since there are limited children's hospitals with pediatric trauma specialists in Japan, severe pediatric trauma patients are often transported to tertiary-care centers for adults [10].

### Japan Trauma Data Bank

The JTDB was established in 2003 by the Japanese Association for Surgery and Trauma (Trauma Surgery Committee) and the Japanese Association for Acute Medicine (Committee for Clinical Care Evaluation) [11]. As of 2018, 280 major trauma care facilities throughout Japan had participated in this registry [12]. Medical staff in participating institutions collected and submitted data using a web-based system and received training in Abbreviated Injury Scale (AIS) coding to ensure the accuracy of registered data. The JTDB steering committee annually distributes pooled data to participating hospitals, following data cleaning and providing feedback to hospitals with significant missing data [13]. The JTDB provides a comprehensive collection of patient information, including age, sex, mechanism of injury, AIS codes (version 1998), Injury Severity Score (ISS), vital signs on hospital arrival, date and time of hospital arrival, procedures (e.g., interventional radiology), surgical operations and computed tomography scans, complications, and mortality at discharge. The ISS is determined by adding the squares of the highest AIS scores in the three most severely injured body regions [14].

### Participants

We included patients with traffic injuries under the age of 18 who were registered in the JTDB with an ISS of at least 16 and who were admitted between 2004 and 2018 were included in this study. We selected an ISS of 16 or higher as the criterion for severe trauma, as it is a widely used cut-off for polytrauma. Patients with an ISS of 16 or higher were more likely to be transported to the tertiary-care hospitals that participated in the JTDB [15]. Patients with missing information on age, sex, or in-hospital mortality, as well as data that were double-counted due to inter-hospital transport, were excluded from the analysis.

### Variables

We collected patient data from the JTDB database, including age, sex, type of injury (blunt or penetrating), time series from the Emergency Medical System call to admission, AIS codes, ISS, vital signs on hospital arrival, and mortality at discharge. To examine seasonal trends, we divided the year into four seasons of three months each: January–March, April–June, July–September, and October–December. We also divided the time of day of the Emergency Medical System call into 6-h intervals (00:00–05:59, 06:00–11:59, 12:00–17:59 and 18:00–23:59). We evaluated injury sites with an AIS score of three or higher in the following body regions: head/neck, thorax, abdomen, pelvis/lower-extremity, and spine. We selected AIS score of three or higher because this AIS cut-off precisely captured clinically defined polytrauma patients [15]. Shock in children was defined as a systolic blood pressure of less than 80 mmHg on hospital arrival, while out-of-hospital cardiac arrest was defined as a systolic blood pressure of 0 mmHg or a heart rate of 0 bpm on hospital arrival [16]. To describe the distribution across the stages of age, we categorized age into four groups: infants/toddlers/preschoolers (0–5 years), middle childhood (6–11 years), young teens (12–14 years), and teenagers (15–17 years) [17].

### Statistical analyses

Continuous variables were presented as the median and interquartile range (IQR), and categorical variables as the number and percentage. To analyze trends in continuous variables, we used the Jonckheere-Terpstra test, while the Cochrane-Armitage test was used to analyze trends in categorical variables. We conducted a multivariable logistic regression analysis to assess factors associated with mortality and calculated adjusted odds ratios (ORs) and 95% confidence intervals (CIs) with a forced entry procedure. The independent parameters included age group, sex, mechanism, season, time of day, injury site, and the 5-year time period, which were selected based on reported findings [18]. We performed subgroup analysis of pediatric traffic injury by car crash and temporal trends. Additionally, we evaluated patient characteristics with pediatric traffic injury by age group using the Kruskal–Wallis test for continuous variables and the chi-squared test for categorical variables. All statistical tests were two-tailed, and we considered *P* values of < 0.05 to indicate statistical significance. We used R Statistical Software (version 3.6.2; R Foundation for Statistical Computing, Vienna, Austria) for all statistical analyses.

## Results

**Fig. 1:**
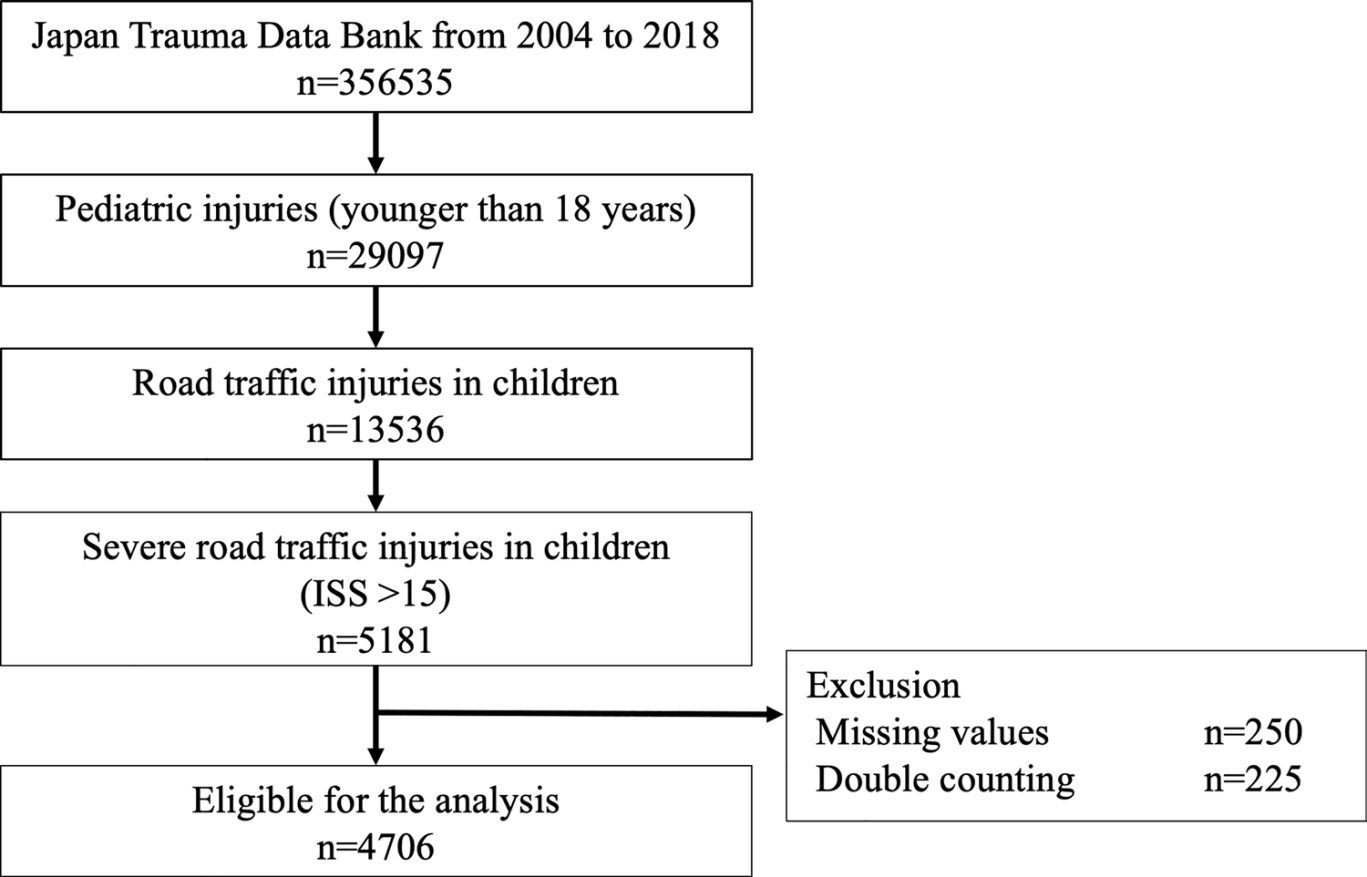
Patient flow

During the study period, 356,535 trauma patients were recorded in the JTDB, of which 29,097 were pediatric trauma patients and 13,536 were pediatric traffic trauma patients. Among them, 5181 were severe traffic trauma patients with ISS > 15, and after excluding missing data and duplicate enrollments, 4706 patients were included in the analysis (Fig. 1).

Table 1 summarizes patient characteristics and their trends over time in this study. Of the 4706 patients included, 771 occurred from 2004 to 2008, 1848 from 2009 to 2013, and 1852 from 2014 to 2018. The median age of all patients was 12 years, and more than half were male (65.9%). Bicycle crashes were the most common mechanism of injury (34.4%), followed by pedestrians (28.3%) and motorcycle crashes (21.3%). Head/neck was the most common injury site (70.7%), followed by thorax (41.8%), pelvis/lower-extremity (15.3%), abdomen (11.8%), and spine (4.0%). The median ISS was 24 (IQR 17–29), 8.6% were in shock on arrival at the hospital, and 5.4% presented with out-of-hospital cardiac arrest. The overall in-hospital mortality was 11.2%. Trends in patient characteristics by 5-year period of admission showed an increasing trend for injuries in rear seat among car crashes and a decreasing trend for motorcycle crashes. Among injury sites, head/neck injury showed a decreasing trend. ISS, shock on arrival, and out-of-hospital cardiac arrest showed also a decreasing trend, and in-hospital mortality also showed a decreasing trend. Patient characteristics by age group are shown in Table S1. Children aged 0–5 years (infants/toddlers/preschoolers) and children aged 6–11 years (middle childhood) were most frequently involved in pedestrians (56.4% and 51.2%), children aged 12–14 years (young teens) were most frequently involved in bicycle crashes (64.0%), and children aged 15–17 years (teenagers) were most frequently involved in motorcycle crashes (53.6%).

**Table 1:** Characteristics of pediatric traffic injury and temporal trends from 2004 to 2018

Characteristics	Total		2004–2008		2009–2013		2014–2018		*p* for Trend
	*n = *4706		*n = *771		*n = *1848		*n = *1852		
Age, median, Q1–Q3	12	8–16	13	8–16	12	8–16	12	8–16	0.752
Age group, *n* (%)									
0–5 years, Infants/toddlers/preschoolers	562	(11.9)	91	(11.8)	245	(13.3)	226	(12.2)	0.956
6–11 years, Middle childhood	1475	(31.3)	258	(33.5)	597	(32.3)	620	(33.5)	0.814
12–14 years, Young teens	662	(14.1)	102	(13.2)	285	(15.4)	275	(14.8)	0.453
15–17 years, Teenagers	1772	(37.7)	320	(41.5)	721	(39.0)	731	(39.5)	0.463
Male sex, *n* (%)	3099	(65.9)	559	(72.5)	1,254	(67.9)	1,286	(69.4)	0.320
Mechanism, *n* (%)									
Car crash	474	(10.1)	68	(8.8)	201	(10.9)	205	(11.1)	0.134
Driver seat	19	(0.4)	1	(0.1)	12	(0.6)	6	(0.3)	0.898
Front passenger seat	144	(3.1)	31	(4.0)	62	(3.4)	51	(2.8)	0.084
Rear passenger seat	311	(6.6)	36	(4.7)	127	(6.9)	148	(8.0)	0.003
Motorcycle crash	1002	(21.3)	220	(28.5)	394	(21.3)	388	(21.0)	< 0.001
Bicycle crash	1620	(34.4)	264	(34.2)	684	(37.0)	672	(36.3)	0.485
Pedestrian	1330	(28.3)	213	(27.6)	551	(29.8)	566	(30.6)	0.157
Unspecified	45	(1.0)	6	(0.8)	18	(1.0)	21	(1.1)	0.396
Season, *n* (%)									
January–March	833	(17.7)	136	(17.6)	349	(18.9)	348	(18.8)	0.575
April–June	1280	(27.2)	228	(29.6)	517	(28.0)	535	(28.9)	0.910
July–September	1300	(27.6)	227	(29.4)	547	(29.6)	526	(28.4)	0.488
October–December	1058	(22.5)	180	(23.3)	435	(23.5)	443	(23.9)	0.728
Time of day, *n* (%)									
00:00–05:59	364	(7.7)	67	(8.7)	151	(8.2)	146	(7.9)	0.498
06:00–11:59	940	(20.0)	148	(19.2)	396	(21.4)	396	(21.4)	0.295
12:00–17:59	2043	(43.4)	358	(46.4)	854	(46.2)	831	(44.9)	0.387
18:00–23:59	1090	(23.2)	188	(24.4)	439	(23.8)	463	(25.0)	0.583
Injury site (AIS 3 +), *n* (%)									
Head/neck	3329	(70.7)	588	(76.3)	1,398	(75.6)	1,343	(72.5)	0.019
Thorax	1967	(41.8)	331	(42.9)	827	(44.8)	809	(43.7)	0.920
Abdomen	557	(11.8)	93	(12.1)	242	(13.1)	222	(12.0)	0.724
Pelvis/lower-extremity	722	(15.3)	136	(17.6)	309	(16.7)	277	(15.0)	0.061
Spine	189	(4.0)	30	(3.9)	74	(4.0)	85	(4.6)	0.341
ISS, median, Q1–Q3	24	17–29	25	18–30	25	17–29	22	17–29	0.004
Shock on arrival, *n* (%)	405	(8.6)	85	(11.0)	176	(9.5)	144	(7.8)	0.005
Out-of-hospital cardiac arrest, *n* (%)	255	(5.4)	66	(8.6)	94	(5.1)	95	(5.1)	0.004
In-hospital mortality, *n* (%)	528	(11.2)	116	(15.0)	231	(12.5)	181	(9.8)	< 0.001

*p* values for trend were calculated using Jonckheere–Terpstra test and Cochrane–Armitage test

*AIS* Abbreviated Injury Scale; *ISS* Injury Severity Score

Table 2 summarizes the association between patient characteristics and in-hospital mortality. After adjusting for age, sex, mechanism of injury, season of injury, time of injury, injury site, and ISS, the adjusted odds ratio (OR) for the association between a 5-year increase in time period and in-hospital mortality was 0.80 (95% confidence interval [CI] 0.70–0.91, *P = *0.001). In-hospital mortality was significantly higher for car crashes, motorcycle crashes, and pedestrians compared to bicycle crashes (adjusted OR 1.69, 95% CI 1.18–2.40, *P = *0.004; 1.42, 95% CI 1.03–1.95, *P = *0.031; 1.50, 95% CI 1.13–1.99, *P = *0.006), and during the hours of 18:00–23:59 and 00:00–05:59 compared to 06:00–11:59 in the morning (adjusted OR 1.37, 95% CI 1.02–1.83, *P = *0.035; 1.77, 95% CI 1.21–2.58, *P = *0.003). Head/neck injuries were most strongly associated with in-hospital mortality among injury sites (adjusted OR 5.06, 95% CI 3.81–6.79, *P < *0.001).

**Table 2:** Odds ratios of each variable for in-hospital mortality among pediatric traffic injury

	Mortality			
	%	*n*/*N*	Adjusted OR (95% CI)	*p* value
Five-year increase in time period	–	–	0.80 (0.70–0.91)	0.001
Age group				
0–5 years, Infants/toddlers/preschoolers	20.3	(114/562)	2.54 (1.73–3.78)	< 0.001
6–11 years, Middle childhood	8.3	(122/1475)	1.02 (0.71–1.48)	0.914
12–14 years, Young teens	8.0	(53/662)	Reference	–
15–17 years, Teenagers	13.5	(239/1772)	1.49 (1.05–2.14)	0.028
Sex				
Male	12.1	(374/3099)	1.03 (0.83–1.28)	0.766
Female	11.2	(154/1372)	Reference	–
Mechanism				
Car crash	16.5	(78/474)	1.69 (1.18–2.40)	0.004
Driver seat	10.5	(2/19)	–	–
Front passenger seat	20.8	(30/144)	–	–
Rear passenger seat	14.8	(46/311)	–	–
Motorcycle crash	15.3	(153/1002)	1.42 (1.03–1.95)	0.031
Bicycle crash	7.3	(119/1620)	Reference	–
Pedestrian	12.8	(170/1330)	1.50 (1.13–1.99)	0.006
Unspecified	17.8	(8/45)	1.90 (0.78–4.14)	0.127
Season				
January–March	11.3	(94/833)	Reference	–
April–June	12.0	(154/1280)	1.15 (0.86–1.53)	0.349
July–September	11.3	(147/1300)	1.00 (0.75–1.33)	0.985
October–December	12.6	(133/1058)	1.16 (0.87–1.56)	0.320
Time of day				
00:00–05:59	18.4	(67/364)	1.77 (1.21–2.58)	0.003
06:00–11:59	10.0	(94/940)	Reference	–
12:00–17:59	10.6	(217/2043)	1.14 (0.87–1.51)	0.335
18:00–23:59	13.6	(148/1090)	1.37 (1.02–1.83)	0.035
Injury site (AIS 3 +)				
Head/neck				
(+)	13.7	(456/3329)	5.06 (3.81–6.79)	< 0.001
(−)	6.3	(72/1142)	Reference	–
Thorax				
(+)	15.6	(307/1967)	2.34 (1.92–2.87)	< 0.001
(−)	8.8	(221/2504)	Reference	–
Abdomen				
(+)	14.2	(79/557)	1.74 (1.29–2.33)	< 0.001
(−)	11.5	(449/3914)	Reference	–
Pelvis/lower-extremity				
(+)	15.9	(115/722)	1.57 (1.23–2.00)	< 0.001
(−)	11.0	(413/3749)	Reference	–
Spine				
(+)	23.3	(44/189)	3.01 (2.02–4.43)	< 0.001
(−)	11.3	(484/4282)	Reference	–

*OR* odds ratio; *CI* confidence interval; *AIS* Abbreviated Injury Scale; *ISS* Injury Severity Score

Table 3 presents patient characteristics and trends over time for pediatric patients with severe traffic injuries in car crashes. There were no differences in trends by age, age group, or sex, but there was a decreasing trend in the passenger seat and an increasing trend in the rear seat. There was also a decreasing trend in time of day from 06:00 to 11:59, and ISS, shock on arrival, out-of-hospital cardiac arrest, and in-hospital mortality showed a decreasing trend. Table S2 shows the association between patient characteristics and in-hospital mortality of pediatric severe traffic injuries in car crashes.

**Table 3:** Characteristics of pediatric traffic injury by car crash and temporal trends from 2004 to 2018

Characteristics	2004–2008		2009–2013		2014–2018		*p* for Trend
	*n = *68		*n = *201		*n = *205		
Age, median, Q1–Q3	7	3–14	8	2–14	9	4–15	0.542
Age group, *n* (%)							
0–5 years, Infants/toddlers/preschoolers	28	(41.2)	77	(38.3)	69	(33.7)	0.207
6–11 years, Middle childhood	16	(23.5)	45	(22.4)	57	(27.8)	0.297
12–14 years, Young teens	7	(10.3)	29	(14.4)	25	(12.2)	0.943
15–17 years, Teenagers	17	(25.0)	50	(24.9)	54	(26.3)	0.761
Male sex, *n* (%)	39	(57.4)	104	(51.7)	118	(57.6)	0.639
Seat position							
Driver seat	1	(1.5)	12	(6.0)	6	(2.9)	0.870
Front passenger seat	31	(45.6)	62	(30.8)	51	(24.9)	0.002
Rear passenger seat	36	(52.9)	127	(63.2)	148	(72.2)	0.002
Season, *n* (%)							
January–March	13	(19.1)	44	(21.9)	42	(20.5)	0.950
April–June	18	(26.5)	40	(20.0)	53	(25.9)	0.652
July–September	24	(35.3)	74	(36.8)	57	(27.8)	0.100
October–December	13	(19.1)	43	(21.4)	53	(25.9)	0.186
Time of day, *n* (%)							
00:00–05:59	9	(13.2)	29	(14.4)	28	(13.7)	0.989
06:00–11:59	23	(33.8)	46	(22.9)	41	(20.0)	0.032
12:00–17:59	23	(33.8)	84	(41.8)	89	(43.4)	0.214
18:00–23:59	11	(16.2)	41	(20.4)	43	(21.0)	0.458
Injury site (AIS 3 +), *n* (%)							
Head/neck	48	(70.6)	145	(72.1)	127	(62.0)	0.059
Thorax	29	(42.6)	95	(47.3)	95	(46.3)	0.723
Abdomen	11	(16.2)	29	(14.4)	30	(14.6)	0.820
Pelvis/lower-extremity	12	(17.6)	27	(13.4)	23	(11.2)	0.179
Spine	6	(8.8)	19	(9.5)	32	(15.6)	0.055
ISS, median, Q1–Q3	25	18–29	22	17–29	22	17–29	0.186
Shock on arrival, *n* (%)	19	(27.9)	36	(17.9)	18	(8.8)	< 0.001
Out-of-hospital cardiac arrest, *n* (%)	14	(20.6)	18	(9.0)	11	(5.4)	< 0.001
In-hospital mortality, *n* (%)	16	(23.5)	41	(20.4)	21	(10.2)	0.002

*p* values for trend were calculated using Jonckheere-Terpstra test and Cochrane-Armitage test

*AIS* Abbreviated Injury Scale; *ISS* Injury Severity Score

## Discussion

This study examined characteristics and trends of severe traffic injuries among children in Japan using data from the JTDB for the past 15 years. Of the 4706 cases analyzed, 771 cases were recorded between 2004 and 2008, which was lower than the 1848 cases registered between 2009 and 2013 and the 1852 cases registered between 2014 and 2018. The JTDB has been in full operation since 2004, and the number of participating facilities has gradually increased from 90 in 2006 to 221 in 2013 and 272 in 2018, which may be one reason for the increase in recorded cases [12, 19, 20]. ISS, shock on arrival, and out-of-hospital cardiac arrest showed a downward trend, as did in-hospital mortality, suggesting that institutions treating severe trauma have been participating since the beginning of JDTB operations, and that from there, the enrollment of less severe trauma may have increased as more facilities participated. However, this study was focused on severe trauma and had approximately the same number of entries from 2009 to 2013 and from 2014 to 2018, suggesting that the epidemiological trends in severe trauma are to some extent accurately reflected in this study.

Bicycle crashes were the most common mechanism of injury, and the trend did not change significantly. Bicycle crashes were more common among young teens aged 12–14 years, presumably because more children use bicycles to get to school, etc. There may be variation in helmet use among school-aged children, and it may be useful to ensure that helmets are used [21]. However, a systematic review found little support for the hypothesis that bicycle helmet use was associated with risky behavior [22]. Curricula that improve knowledge of safe bicycling for school-aged children may also be effective [23]. Nevertheless, a systematic review suggests that bicycle education and skills training programs may increase knowledge of bicycle safety but may not lead to reduced injury rates or improved bicycle operating skills and attitudes [24]. Infrastructure improvements, such as the development of bicycle lanes, may be effective [25]. Other factors may also affect the use of bicycle-mounted child seats on bicycles driven by adults, with reports of bicycle-mounted child seats and baby carriers being dangerous, especially with a high risk of head and neck trauma [26]. Parents using bicycle child seats should use both bicycle helmets and high-back seats and combined use of seat belts is recommended [27]. Since this study did not provide information on the use of helmets or the availability of child safety seats, further research is needed to investigate these factors.

The second most common mechanism of injury was pedestrian crashes, which were associated with in-hospital mortality more than bicycle crashes, although the trend did not change significantly. As previously reported, there is a wide variety of factors associated with pedestrian crashes, including driver, pedestrian, and roadway-related factors, and a combination of measures to address each factor may be effective [28]. In urban areas in particular, pedestrians and traffic volumes are large, and past reports have shown that important risk factors related to pedestrian crash risk include the number of bus stops per unit time, parking spaces, crosswalks, frequency of violations, variations in traffic speed, and number of intersecting side roads, in addition to through and intersecting traffic volumes [29] The design of roadways and the development of various land uses can both increase and decrease pedestrian traffic crash injuries, and urban and regional planners need to design the built environment to minimize pedestrian risk [30]. Widespread use of automatic braking systems to reduce the speed of vehicles in collisions could also reduce serious traffic trauma for pedestrians [31]. Severe traffic trauma while walking was particularly high in this study, especially among 0–5 and 6–11 year-olds, and interventions will have to be made to encourage safe pedestrian behavior in the patients themselves and their families [32].

The third most common mechanism of injury was motorcycle crashes, which, although trending downward, still remain a common cause of severe trauma and are associated with higher in-hospital mortality compared to bicycle crashes. Previous studies have reported that most pediatric motorcycle crashes occurred off-road, but this information was not available in this study [33]. Most pediatric motorcycle crashes tend to result in head and chest injuries, as evidenced by simulator studies [34]. A previous systematic review reported that few studies reported on the effects of interventions, but interventions for adult motorcyclists have shown that they may be applicable to children, and even children would benefit from helmets and protective clothing [35]. The JTDB does not have data on whether or not helmets were worn, making detailed analysis difficult.

For car crashes, which accounted for 10.1% of pediatric severe traffic injuries, there was no overall trend change, but they were associated with higher in-hospital mortality compared to bicycle crashes. Although there was a decreasing trend in front passenger seat injuries, there was an increasing trend in severe injuries in the rear seat. Injuries in the rear seat were more common among children in the age group 0–5 years, indicating the need for countermeasures. Measures to reduce the impact of car crashes mainly include the establishment and implementation of regulations on the use of seat belts and child restraints, advertising campaigns emphasizing the effectiveness of seat belts and child restraints, in-car broadcasts of sound warnings about seat belt use, and the establishment of financing programs and other assistance measures for the purchase of child restraints [36]. In Japan, child car restraints are mandatory for children 6 years old and younger, and 64% of children in Japan use child restraints [37]. Although child restraints are preferred in the rear seat, serious road trauma in the rear seat has shown an increasing trend, and it is important to properly restrain children in appropriate child restraints and to consider the physiological and anatomical specific injury risks due to age and co-factors of road crashes [38]. World Health Organization uses the following criteria to assess legislation on child restraints: Law requires children to use a child seat until at least 10 years or 135 cm tall; Law refers to a standard for child restraints; Law restricts children under a certain age or height from sitting in the front seat [37]. Further dissemination of child restraint systems and their correct use is important. Further research is needed as the JTDB does not have data on whether child restraint systems are installed or not, or whether seat belts are worn or not, making detailed analysis difficult.

National Traffic Safety Campaign in Japan has national priorities that include ensuring safe passage for children and the elderly, promoting safe bicycle use, and ensuring the correct use of seat belts and child restraints in all seats [2]. The most common mechanism of injury in different age groups in the study were pedestrian crashes among children aged 0–5 years and children aged 6–11 years, bicycle crashes among children aged 12–14 years, and motorcycle crashes among children aged 15–17 years. As the mechanism of injury differs according to the developmental stage of the child, road traffic crash prevention measures should be tailored accordingly. Strategies to prevent traffic trauma in children include raising awareness among parents and caregivers, and at the school level after school age [39].

## Limitations

This study has several limitations. First, as mentioned above, JTDB is a hospital-based registry, not a population-based dataset, and thus, it did not include severe traffic injuries that were transported to the hospital not participating in the JTDB, which could bias the results. Given the alteration in participating facility numbers, cautious interpretation of statistical findings is warranted. However, as of March 2018, more than 80% of tertiary-care hospitals across Japan participated in the JTDB registry, implying efficient inclusion of a significant proportion of severe trauma cases in the nation. Furthermore, the resemblance in case counts among the latter two groups across the three 5-year time periods enables a degree of trend comparability. Second, the study did not include patients who were not transported to hospitals, such as unwitnessed trauma victims with rigor mortis, which may lead to an underestimation of the incidence of severe road traffic injuries in this study. Third, the JTDB does not provide information about the causes of crashes, the results of accident investigations, and whether the severely injured children in the backseat were restrained in special seats, which may limit its usefulness as a source of information for developing countermeasures. However, we believe that this study is still important as it provides insights into the situation of severe road traffic injuries in children based on a nationwide database in Japan. Further research is needed to address these limitations.

## Conclusions

This study examined the characteristics and trends of traffic injuries among children using the JTDB and identified factors associated with higher in-hospital mortality such as type of mechanisms and concomitant injuries. The most common mechanism of injury was bicycle crashes, followed by pedestrians and motorcycle crashes, with a decreasing trend in motorcycle crashes. Among car crashes, while injuries in the front passenger seat tended to decrease, those in the rear seat with serious injuries tended to increase. More emphasis must be necessary on child road safety. Strengthening child road safety measures, particularly for rear passenger seats in vehicles, is imperative to enhance our dedication to injury prevention.

## Supplementary Information

Below is the link to the electronic supplementary material.

Supplementary file1 (DOCX 22 KB)

Supplementary file2 (DOCX 21 KB)

## Data Availability

The data that support the findings of this study are available from the JTDB, but the availability of these data is restricted.
