# Supplementary material for: Trends and characteristics of severe road traffic injuries in children: a nationwide cohort study in Japan
Source: Eur J Trauma Emerg Surg. 2023 Oct 17;50(6):2631–40. doi: 10.1007/s00068-023-02372-z (PMC11666704; doi:10.1007/s00068-023-02372-z)
Supplement: Supplementary file 1 — Supplementary file1 (DOCX 22 KB) [file 68_2023_2372_MOESM1_ESM.docx]

**Table S1. Characteristics of patients with pediatric traffic injury by age group**

|  | 0-5 years  Infants/toddlers/ preschoolers | | 6-11 years  Middle childhood | | 12-14 years  Young teens | | 15-17 years  Teenagers | | P value |
| --- | --- | --- | --- | --- | --- | --- | --- | --- | --- |
| Characteristics | n=562 | | n=1,475 | | n=662 | | n=1,772 | |  |
| Age, median, Q1-Q3 | 3 | 2-4 | 8 | 7-10 | 13 | 12-14 | 16 | 16-17 | - |
| Male sex, n (%) | 358 | (63.7) | 986 | (66.8) | 463 | (69.9) | 1,292 | (72.9) | <0.001 |
| Mechanism, n (%) |  |  |  |  |  |  |  |  | <0.001 |
| Car crash | 174 | (31.0) | 118 | (8.0) | 61 | (9.2) | 121 | (6.8) |  |
| Driver seat | 2 | (0.4) | 2 | (0.1) | 3 | (0.5) | 12 | (0.7) |  |
| Front passenger seat | 49 | (8.7) | 24 | (1.6) | 20 | (3.0) | 51 | (2.9) |  |
| Rear passenger seat | 123 | (21.9) | 92 | (6.2) | 38 | (5.7) | 58 | (3.3) |  |
| Motorcycle crash | 4 | (0.7) | 8 | (0.5) | 40 | (6.0) | 950 | (53.6) |  |
| Bicycle crash | 60 | (10.7) | 576 | (39.1) | 424 | (64.0) | 560 | (31.6) |  |
| Pedestrian | 317 | (56.4) | 756 | (51.2) | 133 | (20.1) | 124 | (7.0) |  |
| Unspecified | 7 | (1.2) | 17 | (1.2) | 4 | (0.6) | 17 | (1.0) |  |
| Season, n (%) |  |  |  |  |  |  |  |  | <0.001 |
| January-March | 101 | (18.0) | 228 | (15.5) | 135 | (20.4) | 369 | (20.8) |  |
| April-June | 166 | (29.5) | 479 | (32.5) | 177 | (26.7) | 458 | (25.8) |  |
| July-September | 168 | (29.9) | 411 | (27.9) | 211 | (31.9) | 509 | (28.7) |  |
| October-December | 126 | (22.4) | 357 | (24.2) | 139 | (21.0) | 436 | (24.6) |  |
| Time of day, n (%) |  |  |  |  |  |  |  |  | <0.001 |
| 00:00-05:59 | 11 | (2.0) | 9 | (0.6) | 27 | (4.1) | 317 | (17.9) |  |
| 06:00-11:59 | 137 | (24.4) | 221 | (15.0) | 163 | (24.6) | 419 | (23.6) |  |
| 12:00-17:59 | 287 | (51.1) | 1,040 | (70.5) | 276 | (41.7) | 440 | (24.8) |  |
| 18:00-23:59 | 117 | (20.8) | 191 | (12.9) | 194 | (29.3) | 588 | (33.2) |  |
| Injury site (AIS 3+), n (%) |  |  |  |  |  |  |  |  |  |
| Head/neck | 398 | (70.8) | 1,144 | (77.6) | 543 | (82.0) | 1,244 | (70.2) | <0.001 |
| Thorax | 285 | (50.7) | 589 | (39.9) | 243 | (36.7) | 850 | (48.0) | <0.001 |
| Abdomen | 73 | (13.0) | 159 | (10.8) | 73 | (11.0) | 252 | (14.2) | 0.017 |
| Pelvis/lower-extremity | 61 | (10.9) | 213 | (14.4) | 80 | (12.1) | 368 | (20.8) | <0.001 |
| Spine | 29 | (5.2) | 31 | (2.1) | 25 | (3.8) | 104 | (5.9) | <0.001 |
| ISS, median, Q1-Q3 | 22 | 17-29 | 21 | 17-28 | 24 | 17-29 | 25 | 18-32 | <0.001 |
| Shock on arrival, n (%) | 122 | (21.7) | 96 | (6.5) | 39 | (5.9) | 158 | (8.9) | <0.001 |
| Out-of-hospital cardiac arrest, n (%) | 72 | (12.8) | 64 | (4.3) | 21 | (3.2) | 98 | (5.5) | <0.001 |
| In-hospital mortality, n (%) | 114 | (20.3) | 122 | (8.3) | 53 | (8.0) | 239 | (13.5) | <0.001 |

P values were calculated using Kruskal-Wallis test and chi-squared test.

AIS, Abbreviated Injury Scale; ISS, Injury Severity Score.
