# Supplementary material for: Trends and characteristics of severe road traffic injuries in children: a nationwide cohort study in Japan
Source: Eur J Trauma Emerg Surg. 2023 Oct 17;50(6):2631–40. doi: 10.1007/s00068-023-02372-z (PMC11666704; doi:10.1007/s00068-023-02372-z)
Supplement: Supplementary file 2 — Supplementary file2 (DOCX 21 KB) [file 68_2023_2372_MOESM2_ESM.docx]

**Table S2. Odds ratios of each variable for in-hospital mortality among pediatric traffic injury** **by car crash**

|  | Mortality | |  |  |
| --- | --- | --- | --- | --- |
|  | % | n/N | Adjusted OR (95% CI) | P value |
| Five-year increase in time period | - | - | 0.61 (0.41 to 0.91) | 0.012 |
| Age group |  |  |  |  |
| 0-5 years, Infants/toddlers/preschoolers | 23.6 | (41/174) | 3.30 (1.24 to 10.55) | 0.026 |
| 6-11 years, Middle childhood | 11.9 | (14/118) | 2.02 (0.68 to 6.96) | 0.227 |
| 12-14 years, Young teens | 8.2 | (5/61) | Reference | - |
| 15-17 years, Teenagers | 14.9 | (18/121) | 1.31 (0.42 to 4.60) | 0.652 |
| Sex |  |  |  |  |
| Male | 18.8 | (49/261) | 1.70 (0.98 to 3.02) | 0.063 |
| Female | 13.6 | (29/213) | Reference | - |
| Seat position |  |  |  |  |
| Driver seat | 10.5 | (2/19) | 0.49 (0.07 to 2.21) | 0.405 |
| Front passenger seat | 20.8 | (30/144) | 1.79 (0.98 to 3.25) | 0.056 |
| Rear passenger seat | 14.8 | (46/311) | Reference | - |
| Season |  |  |  |  |
| January-March | 11.1 | (11/99) | Reference | - |
| April-June | 18.0 | (20/111) | 1.84 (0.78 to 4.50) | 0.171 |
| July-September | 14.8 | (23/155) | 1.36 (0.60 to 3.22) | 0.466 |
| October-December | 22.0 | (24/109) | 2.48 (1.09 to 5.96) | 0.035 |
| Time of day |  |  |  |  |
| 00:00-05:59 | 22.7 | (15/66) | 2.83 (1.10 to 7.42) | 0.031 |
| 06:00-11:59 | 16.4 | (18/110) | Reference | - |
| 12:00-17:59 | 13.3 | (26/196) | 1.00 (0.49 to 2.07) | 0.996 |
| 18:00-23:59 | 18.9 | (18/95) | 1.41 (0.64 to 3.13) | 0.393 |
| Injury site (AIS 3+) |  |  |  |  |
| Head/neck |  |  |  |  |
| (+) | 21.6 | (69/320) | 7.84 (3.49 to 19.53) | <0.001 |
| (-) | 5.8 | (9/154) | Reference | - |
| Thorax |  |  |  |  |
| (+) | 17.4 | (38/219) | 2.29 (1.29 to 4.11) | 0.005 |
| (-) | 15.7 | (40/255) | Reference | - |
| Abdomen |  |  |  |  |
| (+) | 10.0 | (7/70) | 0.84 (0.30 to 2.13) | 0.729 |
| (-) | 17.6 | (71/404) | Reference | - |
| Pelvis/lower-extremity |  |  |  |  |
| (+) | 14.5 | (9/62) | 1.22 (0.49 to 2.81) | 0.654 |
| (-) | 16.7 | (69/412) | Reference | - |
| Spine |  |  |  |  |
| (+) | 21.1 | (12/57) | 2.32 (1.01 to 5.33) | 0.048 |
| (-) | 15.8 | (66/417) | Reference | - |

OR, odds ratio; CI, confidence interval; AIS, Abbreviated Injury Scale; ISS, Injury Severity Score.
